# Can a Saddle Nose Deformity of Granulomatosis with Polyangiitis be Repaired?

**DOI:** 10.1007/s00266-017-0888-x

**Published:** 2017-05-10

**Authors:** Osamu Ito, Tomoyuki Yano, Minako Ito

**Affiliations:** Department of Plastic Surgery, Clinical Study Support Center, Medical Check-up Center, Yokohama City Minato Red Cross Hospital, 3-12-1, Shinyamasita, Naka-ku, Yokohama, Kanagawa 231-8682 Japan

*Level of Evidence V* This journal requires that authors assign a level of evidence to each article. For a full description of these Evidence-Based Medicine ratings, please refer to the Table of Contents or the online Instructions to Authors www.springer.com/00266.

We read one review article [1] about saddle nose reconstruction of granulomatosis with polyangiitis (GPA). GPA is a systemic autoimmune disorder that is characterized by both granulomatosis and polyangiitis. Although saddle nose deformity is a common manifestation, appropriate treatment is not adequately reported. So, the article is timely and autocartilage grafts are one of the answers for saddle nose treatments. But our answer to this is not the same. We selected using a silicone implant for a middle-aged woman with GPA.

## Our Case

A 38-year-old woman presented with a right cheek mass, present since the age of 33. She reported bleeding lesions of the limbs, oral ulceration, arthralgia, fever, and disturbance of consciousness from 13 years of age. Repeated swelling in the nose had occurred since she was 18 years old, ultimately resulting in saddle nose deformation. She underwent surgical removal of the tumor (7 × 6 × 5 cm) at 39 years old. Pathologically, her tumor mass granulomas were based on vasculitis and adipose necrosis. She was diagnosed with granulomatosis with polyangiitis: GPA (Wegener’s granulomatosis).

Saddle nose is not only aesthetically unpleasant but results in functional impairment of nasal congestion. We selected surgical reconstruction. Because GPA is an autoimmune disorder, we considered an autograft to be dangerous for the donor and recipient sites. Nasal reconstruction using silicone was considered the best option to treat GPA saddle nose.

The patient underwent surgical reconstruction of the saddle nose using silicone at 40 years old. During the long-term follow-up over 10 years, her general condition was a systemic state of remission with corticosteroid therapy, and her nose condition remained good.

## Discussion

GPA (Wegener’s granulomatosis) [2, 3] is a systemic autoimmune disorder that involves both granulomatosis and polyangiitis. The granulomatosis affects small- and medium-size blood vessels and is formally classified as one of the small vessel vasculitides. Although the buccal masses are rare in GPA [4], this tumor mass was pathologically classical granuloma-based vasculitis and adipose necrosis. GPA has a well-known symptom (saddle nose of the face), and there are few reports of saddle nose therapy with autocartilage [5]. Can a saddle nose deformity of granulomatosis with polyangiitis be repaired? Yes, as long as the underlying vasculitis is not active. Therefore, we selected artificial silicone as a safer material than an autograft for nose reconstruction in autoimmune diseases.
